# Online Consultations Between General Practitioners and Psychiatrists in the Netherlands: A Qualitative Study

**DOI:** 10.3389/fpsyt.2021.775738

**Published:** 2021-11-04

**Authors:** Nynke W. Bock, Hans Wouters, Anne J. Lammers, Marco H. Blanker

**Affiliations:** ^1^Department of General Practice and Elderly Care Medicine, University Medical Centre Groningen, Groningen, Netherlands; ^2^General Practitioners Research Institute, Groningen, Netherlands

**Keywords:** general practice (GP), interdisciplinary consultation, psychiatry, primary care (MeSH), digital

## Abstract

**Objective:** To examine the nature and scope of questions about psychiatric patient cases submitted by general practitioners (GPs) to an established online consultation platform and to determine if they could have been answered by consulting existing clinical guidelines.

**Methods:** All anonymized psychiatric cases submitted by GPs to the online electronic Prisma platform between September 2018 and November 2019 were examined in a mixed-methods study. Descriptive statistics and qualitative thematic analysis were used, followed by axial coding to arrive at overarching themes to characterize cases.

**Results:** Of the 136 included cases, 44.1% concerned female patients and about half concerned patients aged 31–60 years. Common psychiatric disorders were depression, attention deficit hyperactivity disorder, sleeping problems, sexual disorders, and eating disorders. The first response was usually given within 2 h (interquartile range, 0–14.3 h), with 86% answered within 24 h and 95% within 48 h. Qualitative analysis revealed four themes, namely “type of question,” “cases in relation to current clinical guidelines,” “case complexity” and “the doctor being pressured.” Type of question comprised diagnostic, therapeutic, and referral questions. Notably, for 44.1% of questions no current clinical guidelines was present and 46.3% of cases were deemed complex in nature. GPs were willing to share their experiences of coping with being pressured by patients.

**Conclusion:** The findings of this study support the potential for an online electronic consultation platform to facilitate feasible and useful interprofessional consultation between GPs and psychiatrists for a broad range mental illnesses and questions of varying complexity.

## Highlights

- To support Dutch general practitioners (GPs) with providing (mental) health care, a digital interdisciplinary consultation platform has been launched.
- This study supports the potential for this platform to facilitate consultations of psychiatrists by GPs.
- Questions posted by GPs cover a broad range of mental illnesses of varying complexity.
- First responses to questions were rapid with a median time of 2 h.

## Introduction

Psychiatric disorders place a substantial burden on general practitioners (GPs) and other primary health care providers. Previous research has shown that GPs are consulted twice as often by patients with psychiatric problems as other patients (1, 2) and experience greater pressures in these consultations, in part due to a lack of consultation time (1). Moreover, owing to the shift in mental health care provision in the Netherlands from secondary to primary care since 2014, the Dutch GP must now coordinate multidisciplinary care. This has been compounded by an increase in patients seeking help for complex psychiatric problems from 12 to 18% between 2011 and 2017 (3). Taken together, these factors have markedly increased the workload of Dutch GPs. Considering the impediments to adequate and timely referral (4), there is an urgent need to develop feasible, innovative, and clinically useful routes of communication between GPs and psychiatrists.

Intended to support GPs with the various issues surrounding health care provision in primary care, a free and innovative e-health application was introduced in September 2018, called the Prisma platform. Through this, GPs can consult medical specialists, including psychiatrists, by submitting anonymized patient cases with one or more specific queries that they would like to be answered. This consultation takes place asynchronously, meaning that GPs can ask questions and the specialists can answer at a time convenient for both providers, rather than having to respond to a telephone call from the GP who requests consultation. This is akin to other digital interdisciplinary consultation systems that have been introduced elsewhere (5–8). However, a distinct feature of the Prisma platform is that other GPs with access to the platform can read posted cases, to promote learning by other physicians (9). As such, the platform holds promise as a tool for improving both communication and knowledge transfer. However, at the same time it remains unknown whether the platform is suitable for communication about psychiatric cases. Although previous studies have shown that, e-consults decrease the number of referrals, this was with regard to somatic conditions (10, 11). Although electronic consultation services between primary care providers and psychiatry are available elsewhere (12–14), the Prisma platform is the first for Dutch GPs, and has not been evaluated yet. It is possible that psychiatric cases are more difficult to summarize (12, 13, 15), particularly for complex psychiatric problems. In turn, this may act as a barrier to GPs using the system for psychiatric cases associated with the greatest workload.

In this study, we examined the nature and complexity of patient cases submitted by GPs to the Prisma platform for consultation with psychiatrists. We also examined whether the submitted enquiries could have been answered by consulting clinical guidelines for GPs.

## Method

### Study Design

We conducted a mixed-methods study in October 2019, qualitatively reviewing all psychiatric consultation requests submitted by GPs to the Prisma platform since its inception (September 2018). We adopted the “Consolidated Criteria for Reporting Qualitative Research” (COREQ) (16). According to Dutch law, no ethical approval was needed for this study. GP consultations on Prisma take place in accordance with the General Data Protection Regulation (GDPR) of the European Union.

The Prisma platform allows authorized GPs to submit anonymized patient histories with an enquiry. For this, the platform is organized in so called tiles, each with a specific clinical area (e.g., internal medicine, psychiatry, gynecology, dermatology). Specialists, in turn, can request additional information or answer the posted question directly. Other specialists (for the psychiatry tile, this includes psychologists and pharmacists) are also able to comment, and GPs who are not involved in the case can provide feedback based on their own knowledge or experience. All members are encouraged to comment on the feedback provided. By no means can patients utilize the platform.

For the current research, all posts for the psychiatry tile, submitted between September 2018 and October 2019, were exported into an Excel file. Each post contained a user code, a time stamp, and the relevant message content. Cases were bundled using the case number, in order of posts, in a word file. We excluded technical messages in which no case history was posted.

### Qualitative Analysis

Atlas.ti version 8 was used for qualitative thematic analysis, which was performed by two female medical students (NWB and AJL) with theoretical and practical knowledge of psychiatry and primary care. For this, word files of complete cases (from the background information and query posted by the GP to the final answer by a specialist, or other user) were imported in Atlas.ti.

**Figure 1:**
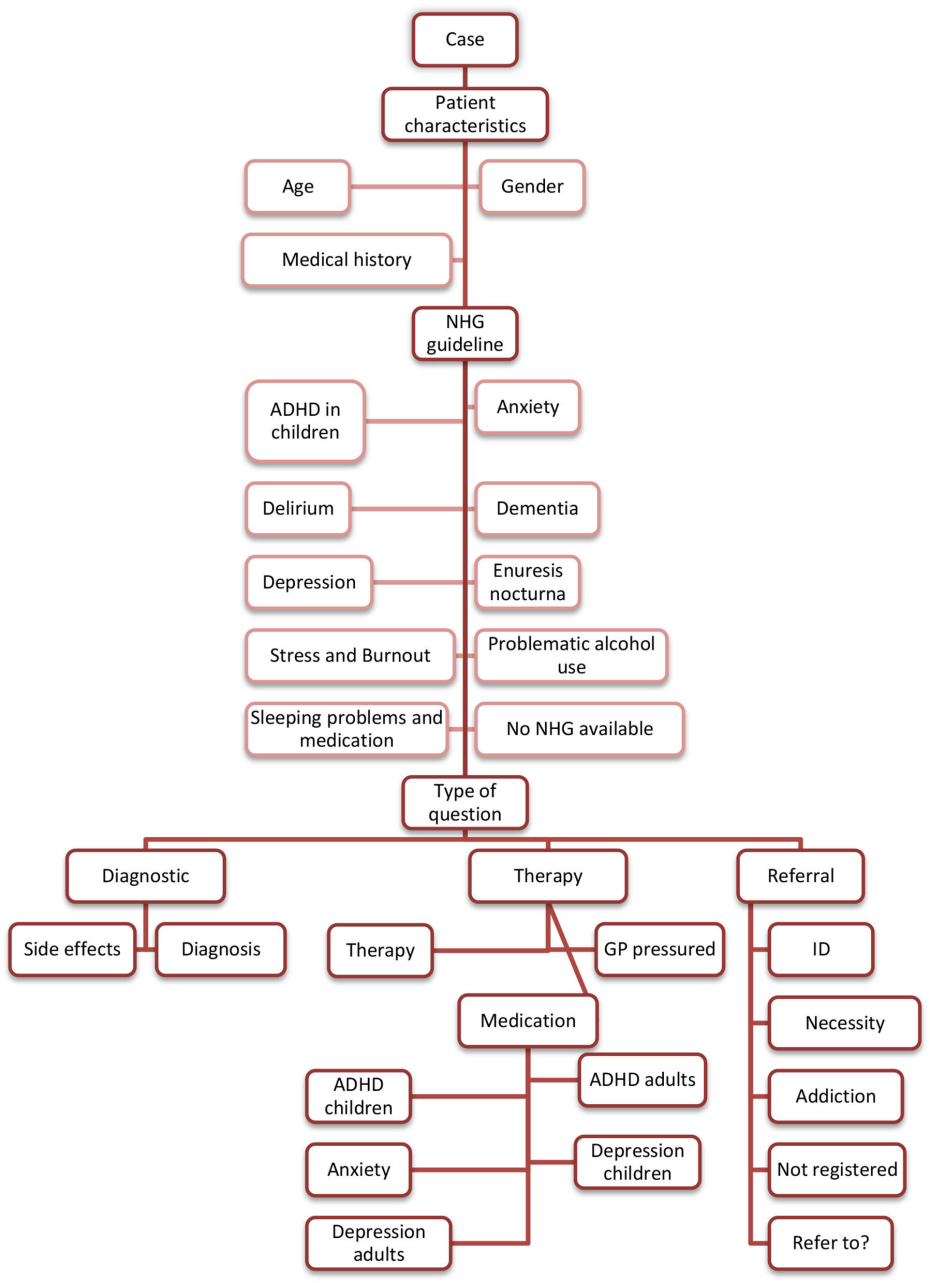
Coding tree. The presented overview does not include codes that did not lead to a theme. ADHD, Attention deficit/hyperactivity disorder; GP, general practitioner; ID, intellectual disability NHG, *Nederlands Huisartsen Genootschap* (Dutch College of General Practitioners).

The researchers started with reading each included case and applying open coding to describe the case backgrounds, and discrepancies in the generated codes were discussed to obtain consensus. The basic coding tree (Figure 1) contained the following themes:

- Nature of the psychiatric problem (i.e., disorders addressed).
- Question type (i.e., diagnosis, therapy, or prognosis).
- Question complexity (i.e., question type, number of advisory steps, number of disciplines involved, number of follow-up questions from the GP, and whether the answer contained advice about communication with the patient). For example: “First reduce drug A, and once reduced, refer the patient.” (See Supplementary File 1).
- Cases in relation to nine clinical guidelines from the Dutch College of General Practitioners (NHG; in Dutch: *Nederlands Huisartsen Genootschap*) guidelines retrieved from https://richtlijnen.nhg.org/ (i.e., attention deficit hyperactivity disorder [ADHD] in children, anxiety, delirium, dementia, depression, nocturnal enuresis, stress and burn-out, problematic alcohol consumption, and sleeping problems and medication).
- For the most common question types, we checked if they could have been answered by consulting the nine NHG guidelines.

To enable reflexivity in the analysis, codes were also regularly reviewed and discussed with two male researchers (HW, a psychologist; MHB, a GP and epidemiologist). Axial coding was subsequently conducted to arrive at overarching themes. Themes that emerged from the data were added to the coding tree as they arose.

### Descriptive Statistics

Code frequency tables for all outcomes were exported from ATLAS.ti to SPSS version 26.0 to provide descriptive statistics. We recorded age, gender, psychiatric disorder, medication, and other relevant data arising from the question (e.g., physical disorders and type of question), describing these as percentages of the total patient population. Age categories were based on the stage of life: childhood (<18 years), young adulthood (19–30 years), middle age (31–60 years), and senior years (>61 years). Based on time stamps and user codes of each post, we estimated the time between question and first answer and the number of users involved in a case. Both were presented as medians with the interquartile range (IQR). We refrained from statistical testing.

## Results

### Participants and Descriptive Statistics

We screened 138 cases for eligibility, of which 1 was a technical message from the support team of the Prisma platform and 1 was an answer to an untraceable question. This left 136 cases for analysis, of which 52.9% concerned females and 41.9% concerned males (5.1% gender not reported), with 44.1% concerning patients aged 31–60 years. The most common diagnoses were depression (19%), ADHD (19%), and sleeping problems (10%), while sexual (2%) and eating (1%) disorders were uncommon.

Cases were posted by 66 different GPs, 2 GP nurses specializing in mental health care (in Dutch: *POH-GGZ*), 1 physician assistant, and 1 rheumatologist. Answers were provided by 12 psychiatrists (1 registrar), supplemented by GP nurses, psychologists, and pharmacists. On average, 3 different users were involved per case (IQR, 2–5). The median time between posting the question and the first response was 2 h (IQR, 0–14.3 h), with 86% answered within 24 h and 95% within 48 h.

Qualitative analysis revealed four themes, namely “type of question,” “cases in relation to current guidelines,” “case complexity,” and “the doctor being pressured.”

### Theme 1: Type of Question

The 136 cases included 169 different questions: 19 (11.2%) diagnostic questions, 113 (66.9%) therapeutic questions, and 37 (21.9%) referral-related questions. A combination of categories was present in 29 (21.3%) cases.

#### Therapeutic Questions

Two therapeutic question types were identified: drug treatment (73.5%, 83 cases) and other treatments (26.5%, 30 cases). “Other treatments” included all forms of therapy, other than medication, such as eye movement desensitization and reprocessing, cognitive behavioral therapy, and lifestyle advice. Some therapeutic questions were formulated broadly, such as “*Do you have any advice on the possible use of palliative psychiatric care?*” (Case 118) concerning a question about palliative care in a psychiatric patient known to have schizophrenia and a metastatic tumor. GPs often asked for advice on psychiatric medication for adults (e.g., converting, reducing, or increasing antidepressant doses), including medication use after a gastric bypass or when there had been a metabolic change.

#### Referral Questions

Questions about referrals included difficulties in deciding if a referral was appropriate.

“[…] *a morbidly obese woman of 32 years with several medical diagnoses … mainly limited by chronic back pain that is untreatable due to obesity. The only treatment options I can think of are psychotherapy, diet counseling, physiotherapy, and occupational therapy. Where could these be* [obtained]*? I've called many obesity clinics* […] *it doesn't seem to exist*.” (Case 126)

Several GPs discussed patients with addiction who were difficult to refer for several reasons. Some were not registered with a GP, while others had allegedly registered at the practice to obtain drug prescriptions under false pretenses in an attempt to maintain their drug addiction.

“*Man, registered in practice with a fabricated story. Turns out he has been addicted for years* … [to different medications, including] … *anything he can get on the black market. Initially* [without knowing of his addiction], *we referred him to mental health care for his ADD and anxiety. He couldn't stay there when the truth was revealed* [.]. *On top of that, we don't want to continue prescribing these amounts of addictive medication. What do we do?”* (Case 86)

Finally, questions were posted about the need for referral, as illustrated by the next quote.

“*Patient has used nortriptyline for 16 years* […]. *currently, there are no signs of depression, but she has had severe panic attacks for a number of years, for which she gets psychotherapy. There is no psychiatrist involved. She would like to reduce the nortriptyline. Can this be done under GP supervision, or does she need the guidance of a psychiatrist?”* (Case 37)

#### Diagnostic Questions

An example of a diagnostic question can be seen in the following presentation of a sleeping problem.

“*Man, 18 years old, suffering attacks of nocturnal restlessness. At 16 years old he had his first attack during the night—he calls it panic attacks, but actually there is no panic. He wakes up, restless, with thoughts of dreams that aren't real. For example, walking on the ceiling. My question: this doesn't sound like panic to me, any idea what it is? Some kind of dream? Sleeping disorder?”* (Case 84)

### Theme 2: Cases in Relation to Clinical Guidelines

**Figure 2:**
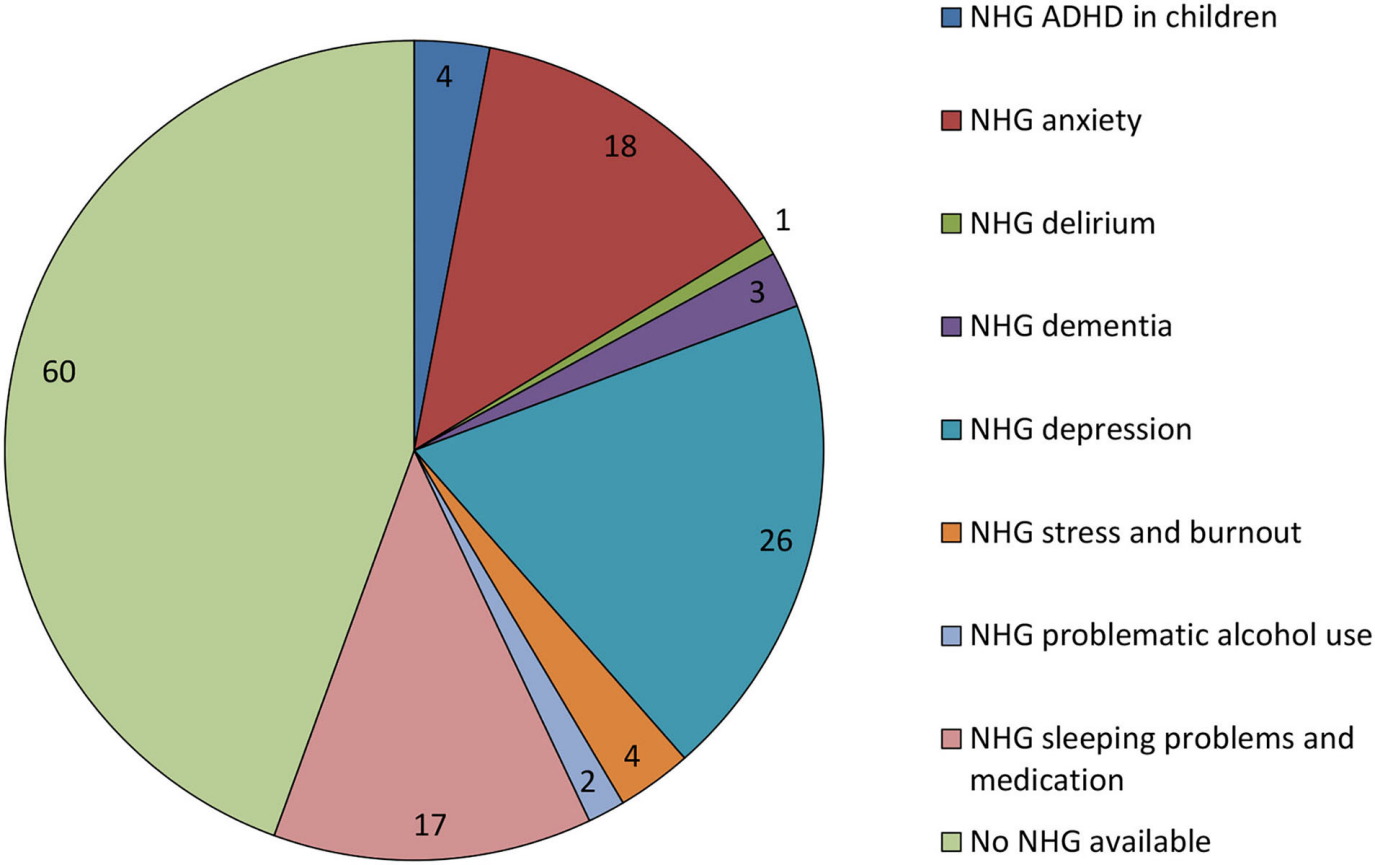
Number of cases with and without NHG guidelines. This figure shows the number of cases with and without guidelines from the Dutch College of General Practitioners (NHG, *Nederlands Huisartsen Genootschap*), including the number for each relevant guideline.

NHG guidelines covered the topic of 76 cases (55.9%) (Figure 2), but no guideline was available for the remaining cases, which included addiction, medical decision-making capacity, ADHD in adults, and eating disorders (Supplementary File 2). Because most questions (*n* = 83) were about medication, we specifically assessed whether these questions could have been answered using the NHG guidelines. However, an NHG guideline could only have answered 11 questions (13.3%) about medication. Examples of those that could not be answered by a guideline included the use of a second-line medication in primary care and when a patient had a complex history. Reasons for cases being considered too complex were the presence of several psychiatric diagnoses for which different treatment methods had already been tried or when there was somatic comorbidity.

### Theme 3: Case Complexity

Complexity was evaluated based on the criteria in Supplementary File 1, with flexibility allowed: although a case could be deemed complex when several steps of advice were given by multiple disciplines, the question itself may not, subjectively, be complex (e.g., “which medication do you recommend”). Overall, 63 (46.3%) and 73 (53.7%) questions were deemed complex and non-complex, respectively. Both reviewers agreed on complexity in 96 cases (70.6%), reached consensus without the aid of a third reviewer in 33 cases, and required the decision of a third reviewer in 7 cases. Complexity did not differ with the presence (36/76 cases) or absence (27/60 cases) of applicable NHG guidelines.

### Theme 4: The Doctor Being Pressured

This coding was applied if a patient had made a dramatic statement, such as “*otherwise I'll kill myself”* (Case 32) or if the GP had made a desperate plea, such as “*what is the best way to deal with this, provide best care and help him* […] *carefully and within the professional care framework?”* (Case 16). In these scenarios, a GP may have felt powerless, even if they knew how to manage the case properly. This category therefore concerned how GPs coped with being pressured by patients. The following cases illustrate this:

“*After visiting him three times this week, he opened his door today*. [He was] *wearing a bathrobe, a bit excited, and said he was alight*. [He] *wished me a pleasant day and closed the door. What can I do, or do I have to do, as a doctor?”* (Case 20)

“*There is a man in my practice who has serious sleeping problems. My predecessor prescribed him Zopiclone. When I became his GP he used 4–6 tablets per night*. […] *The only thing he is prepared to do is take large quantities of Zopiclone because he will sleep and thus function. He has threatened me with ‘*[prescribe it] *or I'll kill myself.'”* (Case 32)

## Discussion

This mixed-method study examined the nature and complexity of psychiatric cases submitted to the Prisma platform for review by a psychiatrist. Cases were fairly well balanced by sex and age, and they covered a variety of psychiatric problems and enquiry types, alike other studies (17, 18). Most cases received a response within 1 day, which is in line with earlier research (15, 17, 19), all showing a seemingly rapid response time. This supports GPs in providing care without a delay, with supportive psychiatric advice. As such, psychiatrists can provide strategies for ongoing management in primary care without an in-person evaluation (12).

These data indicate that the Prisma platform has a case mix that is representative of Dutch primary care practice (20) and that is comparable to earlier research (12, 13). Had the Prisma platform been of less clinical utility, it is unlikely that a broad range of psychiatric problems and important questions (e.g., about treatment) would have been submitted. Beyond this important consideration, three clinical implications warrant discussion.

First, it was not possible to answer the questions with existing clinical guidelines in almost half of the cases. This was even more pronounced for pharmacotherapeutic questions, of which fewer than one in five could have been answered by the NHG guidelines. This may have arisen because the complex nature of the psychological problems for which these guidelines cater increase the likelihood that they will be insufficient. However, it is not realistic for the NHG guidelines to cover all problems that may present to a GP because this would result in them becoming overly complicated. This supports the potential value of the Prisma platform being used as a resource to complement clinical guidelines. We are unaware of studies comparing advices provided through interdisciplinary consultations with current guidelines.

Second, almost half of the cases were deemed objectively complex in nature, supporting the notion that the Prisma platform could be used to ask complex questions. This is reassuring given the potential for practitioners to have reservations about the using Prisma for interprofessional communication about psychiatric problems. Earlier, Canadian primary care providers indicated that psychiatry has a complexity that differentiates it from other specialties and may limit the utility of e-consult, other than for psychopharmacology advice (15). This was illustrated by only a small number of cases being complex in that study. It is unclear if this difference can be explained by cultural differences between the Netherlands and Canada.

Third, when communicating about cases, it was apparent that GPs were willing to describe how they had been pressured by patients. This may provide opportunities for the Prisma platform to be used for peer-to-peer coaching and to support the professional conduct of GPs in difficult cases, and not merely for consultation purposes. Notably, most GPs were unfamiliar with the consulting specialists on the platform. We are unaware if this is a possible facilitator or a barrier for this type of consultations.

The present study benefited from using qualitative thematic analysis to obtain a clear understanding of the cases before using axial coding to arrive at overarching themes, and from using reflexivity during the qualitative coding. However, there are limitations that should also be addressed. First, patients with anxiety disorders were underrepresented compared with the known incidence of anxiety in Dutch primary care (20). It remains unclear if this can be attributed to the comprehensiveness of the GP guideline on anxiety or if GPs considered it less suitable to consult psychiatrists about anxiety, either with or without the Prisma platform. Another limitation was the lack of follow-up information on the impact of advice provided via Prisma. Earlier, a small study showed that primary care providers in the USA implemented the majority [76% (38/50)] of the advices provided by psychiatrists (12). Recently, Avery et al. (17) showed that in 94% (282/300) of psychiatric electronic consultations in a single academic medical center in the UK, at least one advice was implemented.

Follow-up studies are needed to examine the extent and manner to which GPs can incorporate the advice received in the continued care of their patient, including if this improves well-being (21, 22). This should also consider not only if GPs encounter communication problems but also the impact of using Prisma on their practice (e.g., are there associated reductions in the burden of care, the number of referrals, or other indices of health care utilization). Next to this subjective evaluation, the true effectiveness of this platform still needs to be examined because only modest empirical evidence is available for the effectiveness of e-consults on important outcomes (21, 22). Our research group has recently started a stepped-wedge clustered randomized trial to study the effectiveness of the Prisma platform. Additionally, we have performed in-depth interviews with GPs, aimed to clarify the barriers and facilitators for the use of this platform both for somatic and psychiatric cases. Finally, there is scope to investigate the hypothesis that Prisma could serve as a way to educate GPs about, and help to implement, NHG guidelines if key parts of the NHG guidelines are included or linked in answers to submitted patient cases.

In conclusion, our findings support for the utility of the Prisma platform for interdisciplinary consultation between GPs and psychiatrists. The platform is suitable for use in a diverse patient population and is able to cover therapeutic, referral, and diagnostic questions, regardless of their complexity. Although further research is clearly needed, these findings are a first step in showing the feasibility and clinical utility of the Prisma platform.

## Data Availability Statement

The raw data supporting the conclusions of this article will be made available by the authors, without undue reservation.

## Author Contributions

NB, HW, and MB contributed to conception and design of the study. NB and AL performed al data coding. NB wrote the first draft of the manuscript. HW and MB wrote sections of the manuscript. All authors interpreted the data, contributed to manuscript revision, read, and approved the submitted version.

## Funding

The Stichting Stoffels Hornstra funded this study.

## Conflict of Interest

The authors declare that the research was conducted in the absence of any commercial or financial relationships that could be construed as a potential conflict of interest.

## Publisher's Note

All claims expressed in this article are solely those of the authors and do not necessarily represent those of their affiliated organizations, or those of the publisher, the editors and the reviewers. Any product that may be evaluated in this article, or claim that may be made by its manufacturer, is not guaranteed or endorsed by the publisher.

## References

[1] Zantinge EM Verhaak PF Bensing JM. The workload of GPs: patients with psychological and somatic problems compared. Fam Pract. (2005) 22:293–7. 10.1093/fampra/cmh732 15778235

[2] De Sutter M De Sutter A Sundahl N Declercq T Decat P. Inter-professional collaboration reduces the burden of caring for patients with mental illnesses in primary healthcare. a realist evaluation study. Eur J Gen Pract. (2019) 25:236–42. 10.1080/13814788.2019.1640209 31373254 PMC6853250

[3] Increase In Patients With Psychological And Social Problems In General Practice [Dutch] [Toename patiënten met psychische en sociale problemen in de huisartsenpraktijk.] (2018). Available online at: https://www.nivel.nl/nl/nieuws/toename-patienten-met-psychische-en-sociale-problemen-de-huisartsenpraktijk (accessed November 1, 2021).

[4] Olthof M Groenhof F Berger MY. Continuity of care and referral rate: challenges for the future of health care. Fam Pract. (2019) 36:162–5. 10.1093/fampra/cmy048 29860269

[5] Thijssing L van der Heijden JP Chavannes NH Melissant CF Jaspers MW Witkamp L. Telepulmonology: effect on quality and efficiency of care. Respir Med. (2014) 108:314–8. 10.1016/j.rmed.2013.10.017 24210893

[6] van Sinderen F Tensen E van der Heijden JP Witkamp L Jaspers MWM Peute LWP. Is teledermoscopy improving general practitioner skin cancer care? Stud Health Technol Inform. (2019) 264:1795–6. 10.3233/SHTI190652 31438348

[7] van der Velden T Schalk BWM Harmsen M Adriaansens G Schermer TR Ten Dam MA. Implementation of web-based hospital specialist consultations to improve quality and expediency of general practitioners' care: a feasibility study. BMC Fam Pract. (2019) 20:73–5. 10.1186/s12875-019-0960-5 31142267 PMC6540440

[8] Bashshur RL Howell JD Krupinski EA Harms KM Bashshur N Doarn CR. The empirical foundations of telemedicine interventions in primary care. Telemed J E Health. (2016) 22:342–75. 10.1089/tmj.2016.0045 27128779 PMC4860623

[9] Kwok J Olayiwola JN Knox M Murphy EJ Tuot DS. Electronic consultation system demonstrates educational benefit for primary care providers. J Telemed Telecare. (2018) 24:465–72. 10.1177/1357633X17711822 28614974

[10] van der Heijden J P de Keizer NF Bos JD Spuls PI Witkamp L. Teledermatology applied following patient selection by general practitioners in daily practice improves efficiency and quality of care at lower cost. Br J Dermatol. (2011) 165:1058–65. 10.1111/j.1365-2133.2011.10509.x 21729026

[11] Muris D Krekels M Spreeuwenberg A Blom M Bergmans P Cals JWL. General practitioners' use of internal medicine e-consultations. Ned Tijdschr Geneeskd. (2020) 10:164. 32186815

[12] Lowenstein M Bamgbose O Gleason N Feldman MD. Psychiatric Consultation at your fingertips: descriptive analysis of electronic consultation from primary care to psychiatry. J Med Internet Res. (2017) 19:e279. 10.2196/jmir.7921 28778852 PMC5562932

[13] Golberstein E Kolvenbach S Carruthers H Druss B Goering P. Effects of electronic psychiatric consultations on primary care provider perceptions of mental health care: survey results from a randomized evaluation. Healthc). (2018) 6:17–22. 10.1016/j.hjdsi.2017.01.002 28162990

[14] Archibald D Stratton J Liddy C Grant RE Green D Keely EJ. Evaluation of an electronic consultation service in psychiatry for primary care providers. BMC Psychiatry. (2018) 18:119–3. 10.1186/s12888-018-1701-3 29720133 PMC5932827

[15] Hensel JM Yang R Rai M Taylor VH. Optimizing electronic consultation between primary care providers and psychiatrists: mixed-methods study. J Med Internet Res. (2018) 20:e124. 10.2196/jmir.8943 29625949 PMC5910533

[16] Tong A Sainsbury P Craig J. Consolidated criteria for reporting qualitative research (COREQ): a 32-item checklist for interviews and focus groups. Int J Qual Health Care. (2007) 19:349–57. 10.1093/intqhc/mzm042 17872937

[17] Avery J Dwan D Sowden G Duncan M. primary care psychiatry econsults at a rural academic medical center: descriptive analysis. J Med Internet Res. (2021) 23:e24650. 10.2196/24650 34468329 PMC8444033

[18] Zemanek CE Martin KB. Assessing for clinical trends over the first year of a psychiatric electronic consult service. J Acad Consult Liaison Psychiatry. (2021). 10.1016/j.jaclp.2021.09.005. [Epub ahead of print]. 34597854

[19] Ahmed S Kelly YP Behera TR Zelen MH Kuye I Blakey R. Utility, appropriateness, and content of electronic consultations across medical subspecialties. Ann Intern Med. (2020) 172:641–7. 10.7326/M19-3852 32283548

[20] Nielen MMJ Hak K Schermer TRJ. Jaarcijfers aandoeningen: incidenties en prevalenties. Uit: Nivel Zorgregistraties Eerste Lijn. (2020). Available online at: https://www.nivel.nl/nl/nivel-zorgregistraties-eerste-lijn/jaarcijfers-aandoeningen-huisartsenregistraties (accessed November 1, 2021).

[21] Vimalananda VG Orlander JD Afable MK Fincke BG Solch AK Rinne ST. Electronic consultations (E-consults) and their outcomes: a systematic review. J Am Med Inform Assoc. (2020) 27:471–9. 10.1093/jamia/ocz185 31621847 PMC7647247

[22] Gonçalves-Bradley DC J Maria AR Ricci-Cabello I Villanueva G Fønhus MS Glenton C. Mobile technologies to support healthcare provider to healthcare provider communication and management of care. Cochrane Database Syst Rev. (2020) 8:CD012927. 10.1002/14651858.CD012927.pub2 32813281 PMC7437392

