# Supplementary material for: Online Consultations Between General Practitioners and Psychiatrists in the Netherlands: A Qualitative Study
Source: Front Psychiatry. 2021 Nov 4;12:775738. doi: 10.3389/fpsyt.2021.775738 (PMC8600358; doi:10.3389/fpsyt.2021.775738)
Supplement: Supplementary file 1 [file Data_Sheet_1.docx]

**Supplementary File 1: Items used to assess the complexity of cases posted on the Prisma platform**

| Complex | Non-complex |
| --- | --- |
| - GP askes more than one type of question - Answer contains > 1 advice step - Answer was given by > 2 disciplines - Answer was given by < 2 disciplines, but there is a discrepancy in the answers - The answer contains advice on communication - A second question was asked by the GP, or the first question was repeated if the response was insufficient | - GP asks one type of question - Answer contains advice on one subject - The answer was given by > 2 disciplines, but the answer is only confirmed or completed and remains with a single advice |

Note that these distinctions were only considered illustrative when guiding decisions.

Abbreviations: GP, general practitioner.

**Supplementary File 2: Subjects within no available NHG guideline**

| **Subject** | |
| --- | --- |
| Anorexia + low IQ | GHB addiction |
| Asperger’s | Gilles de la Tourette’s syndrome |
| Bipolar disorder | HIV / hepatitis + borderline |
| Bipolar disorder + CBD oil | Homelessness + addiction |
| ADD + depression | Hypochondria + Asperger’s |
| ADHD in adults | Load resilience |
| Autism + Diabetes Mellitus type 1 | Mental disability + anxiety |
| Chronic fatigue | Morbid obesity |
| Confusion | Oxazepam addiction |
| Conversion | Oxycodone withdrawal |
| Decisiveness | Palliative care + schizophrenia |
| Depression child | Psychopharmaceutical addiction |
| Developmental disorder + mood disorder | Schizophrenia |
| Dissociative disorder | Sex addiction |
| Eating disorder + autism | Sexual disinhibition + low IQ |
| Eating disorder general | Sleeping disorder children |
| Euthanasia | Trichotillomania |

Abbreviations: ADD, Attention deficit disorder; ADHD, Attention deficit/hyperactivity disorder; CBD, Cannabidiol; GHB, Gamma Hydroxybutyrate; HIV, Human Immunodeficiency Virus: IQ, intelligence quotient.
